# Supplementary material for: Association between oral microbiome and seven types of cancers in East Asian population: a two-sample Mendelian randomization analysis
Source: Front Mol Biosci. 2023 Nov 21;10:1327893. doi: 10.3389/fmolb.2023.1327893 (PMC10702768; doi:10.3389/fmolb.2023.1327893)
Supplement: Supplementary file 1 [file DataSheet1.ZIP › Supplementary Materials/MR plots of saliva/saliva═╝/Breast cancer/pheno.1539_to_breast cancer_forest.pdf]

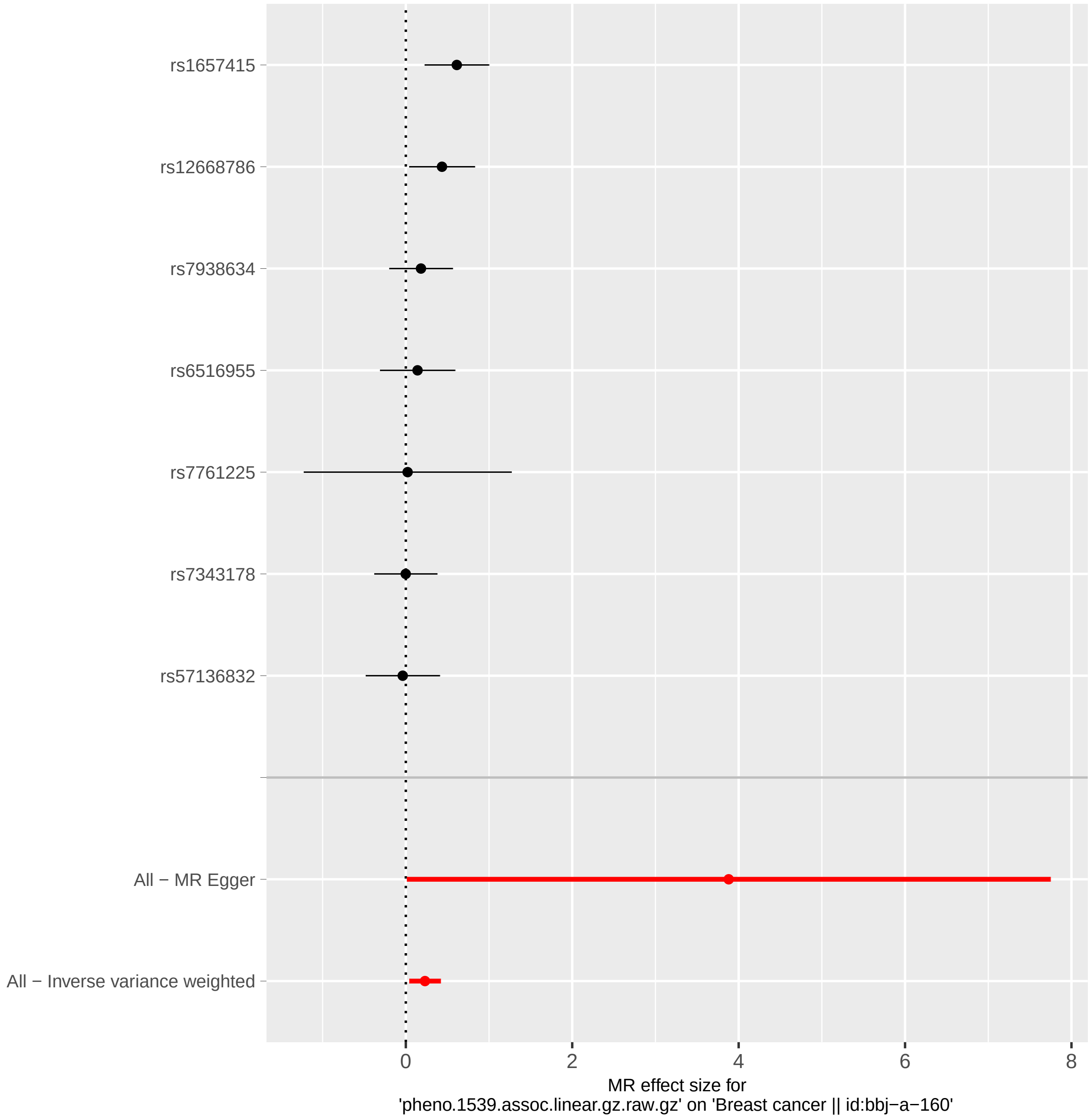
rs1657415
rs12668786
rs7938634
rs6516955
rs7761225
rs7343178
rs57136832
All − MR Egger
All − Inverse variance weighted
0
2
4
6
8
MR effect size for
'pheno.1539.assoc.linear.gz.raw.gz' on 'Breast cancer || id:bbj−a−160'
